# Peritoneal Dialysis and Its Local and Systemic Complications: From the Bench to the Clinic

**DOI:** 10.3389/fphys.2020.00188

**Published:** 2020-02-28

**Authors:** Janusz Witowski, Manuel López-Cabrera

**Affiliations:** ^1^Department of Pathophysiology, Poznan University of Medical Sciences, Poznan, Poland; ^2^Severo Ochoa Molecular Biology Center (CSIC-UAM), Madrid, Spain

**Keywords:** Editorial: peritoneal dialysis, mesothelial cells, renal replacement therapy, peritonitis, peritoneum

Kidney failure is an increasingly common medical problem, posing a serious challenge to health-care systems worldwide and requiring renal replacement therapy at some point. Peritoneal dialysis (PD) is a well-established and cost-effective form of such treatment. It is particularly suitable for home-based therapy and is associated with generally good outcomes and favorable patient experience. However, compared to haemodialysis (HD)—the other dialysis modality—PD is largely underutilized. This can be partly attributed to health care policies but also to limited awareness and availability of the services. Recent experience shows that the improvements in these aspects may increase interest in PD as a treatment option (see Briggs et al., 2019 for a review).

An important problem that hampers PD proliferation is limited technique survival, with only ~50% of patients remaining on PD after 2 years (Mehrotra et al., 2009). The reasons for technique failure can be multiple and of different nature. Among those, adverse remodeling of the peritoneum is a significant problem as it may result in the inability of the peritoneal membrane to sustain effective ultrafiltration and toxin removal. This scenario is often a consequence of peritoneal infection. Thus, peritonitis became one of the key topics and drivers of research in PD. This led to an improved understanding of peritoneal physiology and pathophysiology and produced results whose significance went beyond PD practice alone and contributed to general immunology and pathology. On the other hand, advances in other disciplines opened up new opportunities for improvements in PD.

Despite these substantial implications, PD research is often perceived as a niche that is of no particular interest to non-specialists and practicing physicians. It is therefore of importance that the results of studies on PD are widely and promptly disseminated within the medical community, as this can increase awareness about PD treatment. Thus, the initiative of Frontiers in Physiology to dedicate a special section to problems of PD should be welcomed and appreciated. Here, the reader will find a number of original and review articles devoted to the mechanisms underlying local and systemic complications of PD.

In two articles  Bartosova et al. and Bartosova and Schmitt investigate the effects of new PD fluids, which are considered more biocompatible than conventional ones. Of particular interest is the fact that these were studies of peritoneal biopsies obtained from children undergoing PD, which eliminated the impact of age-related morbidities. These analyses revealed some unexpected morphological features (especially in the peritoneal vasculature), indicating that the effect of new PD fluids is more complex than previously thought. In addition, they showed that the development of peritoneal alterations (at least in a population of pediatric patients) was associated predominantly with the duration of exposure to PD rather than with a history of peritonitis.

Nevertheless, the classic view holds that vascular changes are a hallmark of inflammation. Increasing evidence points to a role for interleukin-17 (IL-17) during inflammation associated with PD. Here, Witowski et al. present the mechanisms by which IL-17 may affect peritoneal vascularity. In turn, Raby and Labeta discuss how toll-like receptors (TLRs) expressed by peritoneal macrophages and mesothelial cells contribute to the course of peritoneal infection. They also highlight the therapeutic implications of inhibiting peritoneal TLRs during peritonitis.

In another article, Krediet a renowned expert in the field of peritoneal transport, discusses how changes in peritoneal vasculature and interstitium lead to impaired fluid transport and ultrafiltration failure during PD. On a similar note, Yu et al. present an original analysis of plasma and dialysate samples from the GLOBAL fluid study. It aimed to determine whether the peritoneal clearance of proteins depended on local or systemic inflammation (Yu et al.). The value of this study is largely that the parameters of local and systemic inflammation, and of solute and protein transport were measured simultaneously (for the first time) in the same large group of well-characterized patients. Peritoneal transport was also evaluated by Olszowska et al., who examined the kinetics of peritoneal ultrafiltration induced by icodextrin. They demonstrate that icodextrin can generate sustained net ultrafiltration even during very long dwells, which—of course—bears important clinical implications for PD patients. The use of Icodextrin-induced ultrafiltration as a treatment option for refractory heart failure was examined by Wojtaszek, Grzejszczak, Niemczyk, et al. The same group looked into the outcomes of PD used as an alternative to HD for patients requiring urgent renal replacement therapy (Wojtaszek, Grzejszczak, Grygiel, et al.).

Other studies focused on metabolic and cardiovascular aspects of PD. As PD patients are exposed to increased loads of glucose from dialysis fluids, Avila-Carrasco et al. examined concentrations of appetite-regulating peptides and eating behavior in relation to patients' body mass and insulin sensitivity. Radunz et al. analyzed more than 4,000 patients from the BRAZPD-II study to determine how glucose exposure impacts on therapy outcomes. They report that higher cumulative glucose exposure does not independently compromise patients' survival, but may be associated with an increased risk of technique failure. Borràs et al. measured the thickness of the carotid intima media in patients from the NEFRONA study assessing the progression of atherosclerosis during kidney disease. Using different models of multivariate analysis, they suggest that the intima in patients on PD may be less thickened than in patients on HD. In turn, Sánchez-González et al. compared patients on PD and HD in terms of parameters related to calcium metabolism. They showed that factors associated with PD affected the diagnostic value of different PTH fragments for metabolic bone disease.

Finally, two articles from the Klaus Kratochwill group showed exciting prospects for the application of proteomics and metabolomics in PD research. These approaches were used to assess the potential of alanyl-glutamine as a cytoprotective additive to PD fluids. This was investigated by proteomic profiling of the peritoneal surface harvested from rats subjected to experimental exposure to PD (Boehm et al.). It was also examined by analyzing the metabolome of the effluent drained from PD patients who were infused with solutions supplemented with alanyl-glutamine (Wiesenhofer et al.).

We believe that the above articles provide a representative selection of studies conducted currently in the field of PD. They should offer good reading for both physicians and basic science researchers.

## Author Contributions

JW and ML-C wrote the editorial.

### Conflict of Interest

The authors declare that the research was conducted in the absence of any commercial or financial relationships that could be construed as a potential conflict of interest.
